# Association among Cognitive Stress Appraisal, Chronic Stress, and Cognition in Cognitively Normal Older Adults: Preliminary Findings

**DOI:** 10.1002/alz.095545

**Published:** 2025-01-09

**Authors:** Paula Aduen, Alex L Badillo‐Cabrera, Clara Vila‐Castelar, Daisy Noriega‐Makarskyy, Diana Munera, John A. Lucas, Clemens Kirschbaum, George M Slavich, Yakeel T. Quiroz

**Affiliations:** ^1^ Mayo Clinic, Jacksonville, FL USA; ^2^ Massachusetts General Hospital, Harvard Medical School, Boston, MA USA; ^3^ University of Southern California, Los Angeles, CA USA; ^4^ Biopsychology, Technische Universität Dresden, Andreas Schubert Bau, Dresden Germany; ^5^ University of California, Riverside, CA USA

## Abstract

**Background:**

Characterizing stress can inform our understanding of direct and indirect pathways impacting mental and physical health outcomes, including age‐related cognitive decline. Chronic stress has been strongly associated with increased risk for cognitive decline, and hair cortisol concentration (HCC) has emerged as a viable neuroendocrine biomarker for its quantification. Cognitive appraisal of stress (CAS) provides key insight into the cognitive framework for responding to a given stressor. We implemented a multi‐pronged approach to evaluate chronic stress and examined its relation to cognition in older adults.

**Method:**

Participants were 22 cognitively normal individuals (x̄ MMSE = 29.33, 0.92; x̄ age: 67.3 ± 8.8 years old, 17.0 ± 2.9 years of education, 54.5% female, 92.6% Non‐Hispanic White). Participants completed the Stress and Adversity Inventory (STRAIN) assessing lifetime stressor exposure and the Primary and Secondary Cognitive Appraisal Scale (PSCAS). HCC was used as a neuroendocrine biomarker of chronic stress. A brief cognitive battery assessing episodic memory and executive functions (Digit Span, HVLT‐R, Trail Making Test, Letter Fluency) and mood/anxiety measures were completed. To correct for skewness, log‐transformed HCC values were used for Pearson and partial correlation analyses controlling for age and education.

**Result:**

Lifetime stressor exposure and CAS were not significantly related to cognitive performance (*all ps* > 0.05). There was a marginally significant association between HCC and executive functions (r = ‐0.41, p = 0.08). Total lifetime stressor count, chronicity, and severity were unrelated to symptoms of depression and anxiety (*all ps* > 0.05). Although lifetime stressor exposure and mood were not significantly related to HCC, higher HCC was associated with higher perceived control of stress (r = 0.45, p = 0.03).

**Conclusion:**

HCC may be more sensitive to CAS rather than to objective stressor exposure. This highlights the potential of interventions targeted at improving coping in those with chronic stress. Results are preliminary, and the present study will explore impact of social and structural determinants of health on chronic stressor exposure, appraisal, and cognition.

